# Combination of LIGHT (TNFSF14)-Armed Myxoma Virus Pre-Loaded into ADSCs and Gemcitabine in the Treatment of Experimental Orthotopic Murine Pancreatic Adenocarcinoma

**DOI:** 10.3390/cancers14082022

**Published:** 2022-04-16

**Authors:** Joanna Jazowiecka-Rakus, Aleksander Sochanik, Agata Hadryś, Wojciech Fidyk, Ewa Chmielik, Masmudur M. Rahman, Grant McFadden

**Affiliations:** 1 Center for Translational Research and Molecular Biology of Cancer, Maria Skłodowska-Curie National Research Institute of Oncology, Gliwice Branch, Wybrzeże AK 15, 44-102 Gliwice, Poland; aleksander.sochanik@io.gliwice.pl (A.S.); agata.hadrys@io.gliwice.pl (A.H.); 2 Department of Bone Marrow Transplantation and Hematology-Oncology, Maria Skłodowska-Curie National Research Institute of Oncology, Gliwice Branch, Wybrzeże AK 15, 44-102 Gliwice, Poland; wojciech.fidyk@io.gliwice.pl; 3 Tumor Pathology Department, Maria Skłodowska-Curie National Research Institute of Oncology, Gliwice Branch, Wybrzeże AK 15, 44-102 Gliwice, Poland; ewa.chmielik@io.gliwice.pl; 4 Biodesign Institute, Arizona State University, Tempe, AZ 85287, USA; masmudur.rahman@asu.edu (M.M.R.); grantmcf@asu.edu (G.M.)

**Keywords:** adipose tissue-derived stem cells (ADSCs), oncolytic virus, myxoma virus, oncolytic virotherapy, pancreatic ductal adenocarcinoma, immune response, gemcitabine

## Abstract

**Simple Summary:**

Pancreatic ductal adenocarcinoma (PDAC) is a weakly immunogenic fatal neoplasm. Oncolytic viruses have dual anti-cancer properties including tumor-lysing and immune response-boosting effects and offer attractive alternative for PDAC management. Adipose-derived stem cells (AD-SCs) of mesenchymal origin were infected ex vivo with recombinant oncolytic myxoma virus (MYXV), which encodes murine LIGHT, also called tumor necrosis factor ligand superfamily member 14 (TNFSF14). ADSC-shielded virus were administered into murine pancreatic cancer lesions that had been induced orthotopically in immunocompetent mice. Ensuing oncolysis and the activation of anti-tumor immune responses provided significant survival benefit. Although adjunct therapy with gemcitabine improved the cytolytic killing of pancreatic cancer cells in vitro, it induced no additional survival advantage in this model in vivo.

**Abstract:**

Pancreatic ductal adenocarcinoma (PDAC) is a deadly neoplasm. Oncolytic viruses have tumorolytic and immune response-boosting effects and present great potential for PDAC management. We used LIGHT-armed myxoma virus (vMyx-LIGHT) loaded ex vivo into human adipose-derived mesenchymal stem cells (ADSCs) to evaluate murine PDAC treatment in conjunction with gemcitabine (GEM). The cytotoxicity of this treatment was confirmed in vitro using human and murine pancreatic cancer cell cultures, which were more sensitive to the combined approach and largely destroyed. Unlike cancer cells, ADSCs sustain significant viability after infection. The in vivo administration of vMyx-LIGHT-loaded ADSCs and gemcitabine was evaluated using immunocompetent mice with induced orthotopic PDAC lesions. The expression of virus-encoded LIGHT increased the influx of T cells to the tumor site. Shielded virus followed by gemcitabine improved tumor regression and survival. The addition of gemcitabine slightly compromised the adaptive immune response boost obtained with the shielded virus alone, conferring no survival benefit. ADSCs pre-loaded with vMyx-LIGHT allowed the effective transport of the oncolytic construct to PDAC lesions and yielded significant immune response; additional GEM administration failed to improve survival. In view of our results, the delivery of targeted/shielded virus in combination with TGF-β ablation and/or checkpoint inhibitors is a promising option to improve the therapeutic effects of vMyx-LIGHT/ADSCs against PDAC in vivo.

## 1. Introduction

Pancreatic cancer is a highly aggressive malignancy characterized by lack of specific symptoms in the early stages, rapid progression, invasiveness and resistance to treatment. It is currently the fifth leading cause of cancer-related death in the world and, due to increasing incidence, is expected to rank second by 2030 [1]. Pancreatic ductal adenocarcinomas (PDAC) which affect the digestive enzyme-producing exocrine part of the organ make up 95% of cases, leading to half a million deaths worldwide every year. Currently, the 5-year survival rate in pancreatic cancer is below 10%, and the median survival from diagnosis is approximately 6 months [1]. Surgery, chemo- and radiotherapy are not likely to improve these grim statistics. Most patients present at unresectable or even metastatic disease stage [2], and unfavorable attributes of PDAC, such as poor vascularity and dense fibroblastic stroma (desmoplasia), attenuate drug delivery, making the clinical outcome of chemotherapy largely ineffective. Some survival improvement is attainable for surgery with chemotherapy but only in early-stage disease, and radiotherapy response is limited due to early metastases. Immunotherapeutic strategies, such as the use of checkpoint inhibitors in PDAC, have not been satisfying either, presumably due to the nonimmunogenic and immune-suppressive tumor microenvironment of PDAC [2]. This grim reality of PDAC [3] resistance to treatment is rooted in key mutations, genetic heterogeneity, the presence of tumor-initiating stem cells and multiple signaling pathway modifications [4]. 

Efficacious PDAC therapy appears to demand the coordinated implementation of agents able to overcome PDAC desmoplasia and trigger potent and long-lasting anti-cancer immunity. This stipulation seems to be met by oncolytic virotherapy, a relatively novel anticancer tool. 

Oncolytic virotherapy provides an innovative immunomodulatory strategy as a new treatment option for a variety of cancers [5]. Among them, the herpes simplex viral construct (Talimogene laherparepvec, T-VEC) approved for patients with advanced melanoma in 2015. Therefore, oncolytic virotherapy is becoming increasingly popular in the treatment of many different forms of cancer, including pancreatic cancer. Oncolytic viruses can yield the disintegration of the targeted tumor fabric (hence oncolysis) following the immunogenic death (ICD) of targeted cancer cells and elicit antitumor immune response. Due to this double-edged capacity, oncolytic viruses seem suitable for adjunct combinations with immuno- or chemotherapeutic agents, yielding a powerful tool for anti-PDAC strategy.

Here, we made use of the oncolytic myxoma virus (MYXV) [6], a type of poxvirus that causes myxomatosis disease in rabbits but is harmless to humans as well as rodents [7]. The permissiveness of most human (and murine) cancer cells to productive infection has allowed the deployment of MYXV as an anticancer therapy vector [8]. The large and stable MYXV genome also allows it to be armed with various therapeutic transgenes via genetic recombination [9]. Such modal constructs can be powerful tools against various neoplasms [10,11,12,13]. 

The MYXV construct used herein was armed with the murine LIGHT gene, whose expressed product is a protein that encodes member 14 of the mouse tumor necrosis factor ligand superfamily (Tnfsf14). It was designed to increase the influx of T lymphocytes and NK cells to the tumor site. LIGHT is believed to act against cancer cells via a combination of two mechanisms: the ability to stimulate antiviral T-cell proliferation [14] and activation of the immune system to stimulate tumor-specific memory T-cell responses [14,15,16]. This LIGHT-armed MYXV construct was first reported in our previous study in which it was employed for the oncoviral monotherapy of experimental PDAC lesions in immunocompetent mice [11]. 

In the present study, we wanted to check whether this LIGHT-armed virus construct would achieve a positive effect in PDAC treatment when combined with gemcitabine chemotherapy. Wennier et al. [17] showed that wild-type myxoma virus did exert a synergistic therapeutic effect with gemcitabine. Such synergy in animal models had also been previously noted for herpersviruses and parvoviruses [17], and recently was reported for vaccinia virus [18]. Unlike Wennier et al., who used a disseminated PDAC model, we pursued treatment in a much more challenging orthotopic PDAC model, which features desmoplasia. 

Gemcitabine (GEM), a pyrimidine nucleoside antimetabolite, once incorporated into DNA, inhibits chain elongation. GEM can also induce reactive oxygen species, block the cell cycle and trigger apoptosis in pancreatic carcinoma cells by lowering the expression of Bcl-2 and activating caspases [19]. The reported benefits of GEM include a reduction in T_reg_ activity, an increase in NK cell activity and the production of IL-12, as well as induced changes in the CTL/T_reg_ ratio in the tumor microenvironment. Administered gemcitabine is prone to rapid clearance due to its short half-life (ca. 20 min.) and deamination of gemcitabine triphosphate to inactive metabolite in blood, liver and kidney [20]. 

The poor vascularization of PDAC, desmoplasia and antiviral immune response of the competent host discourage the intravenous administration of unshielded virus. The oncoviral therapeutic was thus shielded with a “Trojan horse” carrier [21]. To avoid the “first pass” effect of virus-laden cellular carrier transit through the lungs [10,11], the therapeutic oncolytic payload was effectively delivered to PDAC lesions induced in mice using infected adipose-derived mesenchymal stem cells (ADSCs) administered via intraperitoneal (ip.) injection. We previously showed the feasibility and effectiveness of delivering the LIGHT-expressing MYXV construct to pancreatic PDAC lesions [11]. 

Unlike Wennier et al. [17], who investigated MYXV and GEM combination using the late-stage intraperitoneal-disseminated (IPD) tumor model, we here examined a novel, much more demanding approach based on orthotopic PDAC lesions surgically induced in the pancreata of immunocompetent mice. The novel model better imitates conditions encountered during actual combination PDAC therapy attempts. The additional challenge is mainly due to the presence of the desmoplastic tumor stroma, which makes the outcome of combination treatment unpredictable. 

We first assessed the expression of therapeutic LIGHT protein using a panel of human and murine pancreatic cancer cell lines infected with the LIGHT-armed construct. Next, we examined and compared the viability of cell cultures treated with the oncolytic construct, or GEM, or both. Finally, we studied the therapeutic effect of ADSC-shielded LIGHT-armed myxoma construct and GEM on immunocompetent mice bearing induced lesions. Using flow cytometry and RT-qPCR, we examined differences in the triggered immune response, the expression of some immune response-related genes and the histopathology of pancreata based on the curative outcome of LIGHT-armed MYXV in combination with GEM.

## 2. Materials and Methods

### 2.1. Cell Culture 

Methods used for acquiring/culturing human ADSCs, rabbit kidney RK13 and pancreatic ductal adenocarcionoma (PDAC) cell lines were described in Jazowiecka-Rakus et al., 2021 [11]. ADSCs were obtained from human fat samples and were used to transport the viral construct. vMyx-LIGHT was propagated in rabbit cell line RK13 (ATCC), which is fully permissive to viral infection. The murine PDAC line (Pan02 and Pan02-luc) was originally a gift from E. Scott (University of Florida, Gainesville, FL) to G. McFadden. It is a non-metastatic cell line sensitive to gemcitabine, a drug used as a first-line treatment for pancreatic cancer. Human PDAC lines (AsPC-1 and Panc-1) were from ATCC. Both lines harbor mutant K-Ras gene implicated in the EGFR pathway. Cell cultures were maintained at 37 °C in a humidified 5% CO_2_ incubator using 10% FBS-supplemented tissue culture media with antibiotics. RK13, Panc-1, Pan02 and Pan02-luc cells were maintained in DMEM and AsPC-1 in RPMI-1640.

### 2.2. Recombinant Virus, Purification and Titration

vMyx-mLIGHT-FLuc/tdTr recombinant myxoma construct, abbreviated here as vMyx-LIGHT, was used. It is derived from the wild-type Lausanne strain of myxoma virus (vMyx-WT). The recombination cassettes were inserted into the intergenic region between the M135 and M136 open reading frames (ORFs). The vMyx-LIGHT construct expresses murine LIGHT and FLuc (firefly luciferase) at both the early and late infection stages (early/late promoter) and tdTr (tandem dimer Tomato red fluorescent protein) under a synthetic poxvirus late promoter. Details of vMyx-mLIGHT-FLuc/tdTr construction were reported in [11,22]. The vMyx-LIGHT recombinant construct was produced and purified as described previously [11,23]. In brief, RK13 cells were infected with the virus (MOI = 0.1). When the cytopathic effect was visible (ca. 72 h), cells were harvested by scraping, freeze–thawed and sonicated. Homogenates were ultracentrifuged using a sucrose cushion. Viral particles in the pellet were titered by serial dilution, and red fluorescent foci were counted.

### 2.3. Susceptibility to Infection with vMyx-LIGHT

The tested cell lines were examined for susceptibility to infection with vMyx-LIGHT and the presence of late gene expression. Briefly, cell cultures were established in chamber slides and infected with vMyx-LIGHT. Following overnight incubation, cells were fixed in paraformaldehyde and examined using confocal fluorescence microscopy (Zeiss LSM 710 workstation). Details are provided in [11].

### 2.4. Quantitative Analysis of Apoptosis by Flow Cytometry 

Cultured cells were seeded at a density of 1 × 10^5^ cells/well using a 6-well plate, and then vMyx-mLIGHT-FLuc/tdTr (MOI = 5) was added to the cells. After 24 h and 48 h, the cells were collected and washed twice with PBS¯ and staining buffer. Then, the cells were stained with anti-Annexin V antibody and 7-aminoactinomycin D (BioLegend, San Diego, CA, USA; kit # 640922) and analyzed for apoptosis/necrosis using flow cytometry (BD FACS Canto II). Annexin V was detected using an FITC channel and 7-AAD using a PerCP-Cy5.5 channel, and a region for live cells was defined. Non-infected cells were used as a control.

### 2.5. Cytotoxicity of vMyx-LIGHT and Gemcitabine 

Cell cultures (ADSCs, Pan02, Panc-1, AsPC-1) were maintained at 37 °C in a humidified 5% CO^2^ incubator using 10% FBS-supplemented tissue culture media with antibiotics. To determine gemcitabine toxicity, cultured cells (1 × 10^4^/well; 96-well plate) were treated with GEM (Accord Healthcare, Poland) at various concentrations (1 nM, 10 nM, 100 nM, 1 µM, 10 µM, 50 µM, 100 µM, 500 µM and 1 mM) for 24, 48 and 72 h. Cell viability was evaluated using MTS assay (CellTiter 96^®^ AQueous Non-Radioactive Cell Proliferation Assay kit; Promega, Poland) and a Biotek plate reader (490 nm). To assess the cytotoxic effects of the GEM and MYXV combination, the cell cultures were infected with vMyx-mLIGHT-Fluc/tdTr at MOI = 5. After 24 h, GEM was added using three concentrations: the one generating the maximum effect at 72 h and two lower values (10 µM, 1 µM and 100 nM, respectively), and cell viability was evaluated after 24, 48 and 72 h using MTS assay as above. Non-infected cells were used as the control. The assays were performed in triplicate.

### 2.6. Animal Care

Female C57Bl/6NCrl immunocompetent mice purchased from Charles River Laboratories were used at 6–8 weeks of age. Animals were housed in the Animal Facility under sterile conditions using individually ventilated cages (Allentown Caging Equipment, AnimaLab, Poland) under a controlled 12 h/12 h light:dark cycle and had free access to a pathogen-free Altromin 1314 standard diet and water. All husbandry procedures and experiments were carried out in accordance with European Union law and institutional standards. All efforts were made to minimize animal suffering. Study approval was obtained from the Local Ethics Committee for Animal Testing, Medical University of Silesia, Katowice, Poland (Approval No. 18/2021). Tumors were established in recipient mice on day 0 via abdominal surgery and an orthotopic pancreas injection of 30 µL PBS¯ containing 1 × 10^6^ Pan02 cells. The mice were then assigned into treatment groups (*n* = 9 or 12, depending on group).

### 2.7. Therapeutic Treatments with Oncolytic Virus or/and Gemcitabine

For oncolytic treatment, animals with pancreatic tumors were injected ip. with vMyx-LIGHT or with vMyx-LIGHT after virus pre-loading ex vivo (MOI = 5) onto ADSCs for 24 h, as previously described [11]. Briefly, the mice were administered the unshielded (2.5 × 10^6^ FFU/100 µL PBS¯) or shielded oncolytic virus (5 × 10^5^ cells/100 µL PBS¯) five times, every second day starting on day 4. For combination therapy with gemcitabine, mice treated as above were additionally given three doses of the chemotherapy (66.5 mg/kg in 100 µL PBS¯) every three days, starting on day 14. 

On days 14 and 21 of treatment (i.e., after the conclusion of oncolytic therapy arm and gemcitabine administration, respectively), some of the animals (*n* = 3) in each study group was sacrificed to collect pancreas, spleen and liver tissues, as well as peripheral blood aliquots. Pancreatic and blood samples were further processed for subsequent flow cytometry and RT-qPCR analyses. Pancreas, spleen and liver tissues were also fixed in formalin; paraffin-embedded pancreas sections (5 µm thick) were H&E stained, scanned using a digital slide scanner (3DHISTECH Ltd.) and analyzed microscopically using CaseCenter 2.9 SP1 software (3DHISTECH Ltd.) by an experienced pathologist.

The remnant mice undergoing treatment (*n* = 6) were observed for survival.

### 2.8. Flow Cytometric Analysis of Tumor-Infiltrating Lymphocytes 

The analysis was carried out as described previously [11]. In brief, the enzymatically digested (collagenase, hyaluronidase type IV-S and DNase I) pancreatic tissue samples (*n* = 3) were mashed through nylon mesh cell strainers (70 µm). Single-cell sample suspensions devoid of red blood cells were treated for 20 min/RT with antibodies, fluorescently labeled and analyzed (no less than 4 × 10^4^ cells) using flow cytometry (BD FACS Canto II) to quantify the percentage of CD4+ and CD8+ populations in pancreas and blood specimens. Antibodies (BioLegend, San Diego, CA, USA) were used according to the manufacturer’s instructions: PerCP/Cyanine5.5 anti-mouse CD45 (clone 30-F11), phycoerythrin (PE) anti-mouse CD3 (clone 17A2), fluorescein isothiocyanate (FITC) anti-mouse CD4 (clone GK1.5) and APC/Cyanine7 anti-mouse CD8a (clone 53-6.7). 

### 2.9. RNA Isolation, cDNA Synthesis and RT-qPCR

Total RNA was isolated from cultures of ADSCs, as well as Pan02, Panc-1 and AsPC-1 pancreatic adenocarcinoma cell lines (5 × 10^5^ cells/flask) infected with vMyx-LIGHT (MOI = 5), as well as from intact pancreata (*n* = 3) using an RNeasy Mini Kit (Qiagen, Poland) according to the manufacturer’s instructions. The synthesis of cDNA was performed using the High-Capacity cDNA Reverse Transcription Kit (Applied Biosystems, Poland), as described previously in detail [8]. RT-qPCR reactions were performed in duplicate for each sample. For primers, see Supplementary Materials, Table S1. For each run, melt curve analysis was performed. The relative quantification of the gene expression level was determined based on the Pfaffl method [24].

### 2.10. In Vivo Bioluminescence Imaging

Tumor growth dynamics were assessed using IVIS Lumina II and Living Image 3.2 software (PerkinElmer, Poland). Animals were injected ip. with d-luciferin (15 mg/mL; VivoGlo Luciferin; Promega, Poland) suspended in 200 μL PBS¯ and were then sedated using isoflurane (2%). 

### 2.11. Statistics 

Graphs were plotted and the analysis of statistical differences performed using GraphPad Prism software (Version 7). The results were analyzed using a one- or two-way ANOVA test followed by Tukey’s multiple comparison test. To ensure that the data met the assumptions of parametric significance tests, Bartlett’s test was run. Kaplan–Meier survival curves were determined using the Mantel–Cox test. Data are presented as bars with mean ± standard deviation. The levels of significance are indicated as follows: * *p* ≤ 0.05; ** *p* ≤ 0.01; *** *p* ≤ 0.001). *p*-values below 0.05 were judged as statistically significant.

## 3. Results

### 3.1. Replication of vMyx-LIGHT in PDAC Cell Lines

**Figure 1:**
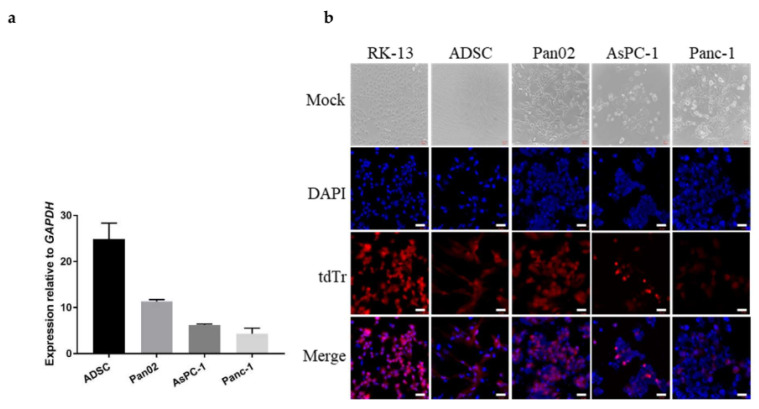
Early and late gene expression following infection of various cell lines with LIGHT-encoding myxoma construct. Cultures of ADSCs, Pan02, RK13, AsPC-1 and Panc-1 were infected with vMyx-mLIGHT-FLuc/tdTr (MOI = 5). (**a**) Constitutive expression of LIGHT gene in infected ADSCs and pancreatic cancer cell lines at 24 h p.i. LIGHT gene transcript was measured using RT-qPCR, and expression was rendered as a ratio of target gene (LIGHT) vs. reference gene (glyceraldehyde 3-phosphate dehydrogenase /*GAPDH*/). The data show mean ± SD of two independent experiments. (**b**) Infection visualized at 24 h p.i. by fluorescence microscopy (magn. 20×; scale bar = 50 µm; Zeiss LSM 710 confocal Workstation); blue: DAPI staining (nuclei); red: tdTr fluorescence.

Three pancreatic ductal adenocarcinoma cell lines were tested for permissiveness to infection with vMyx-LIGHT. Adipose-derived stem cells, used here as a carrier for the oncolytic construct and rabbit RK13 cells in which myxoma virus is easily propagated, were used as controls. LIGHT and Fluc showed early and late expression, whereas tdTr showed late expression only. First, we confirmed the expression of the LIGHT gene in ADSCs and the three pancreatic cancer cell lines examined 24 h postinfection with vMyx-LIGHT (Figure 1a). LIGHT expression in ADSCs was twice as high as in murine Pan02. The lowest expression of LIGHT was found for the two human pancreatic cancer cell lines studied.

The analysis of fluorescence microscopy images revealed differences in the late protein (tdTr) expression among the tested cell types (Figure 1b). In ADSC, RK13 and murine Pan02 cell cultures, cell-to-cell spread and permissive infection were observed. Human PDAC lines (AsPc-1 and Panc-1) appeared to be infected to a lesser degree and can be considered semi-permissive for MYXV replication, with at least detectable levels of LIGHT gene expression (see Figure 1a). In sum, the different types of pancreatic cancer cells tested have varying permissiveness for the vMyx-LIGHT construct, but they support observable levels of early virus gene expression.

### 3.2. Determination of Apoptosis and Necrosis after the Treatment of Cells with vMyx-LIGHT

**Figure 2:**
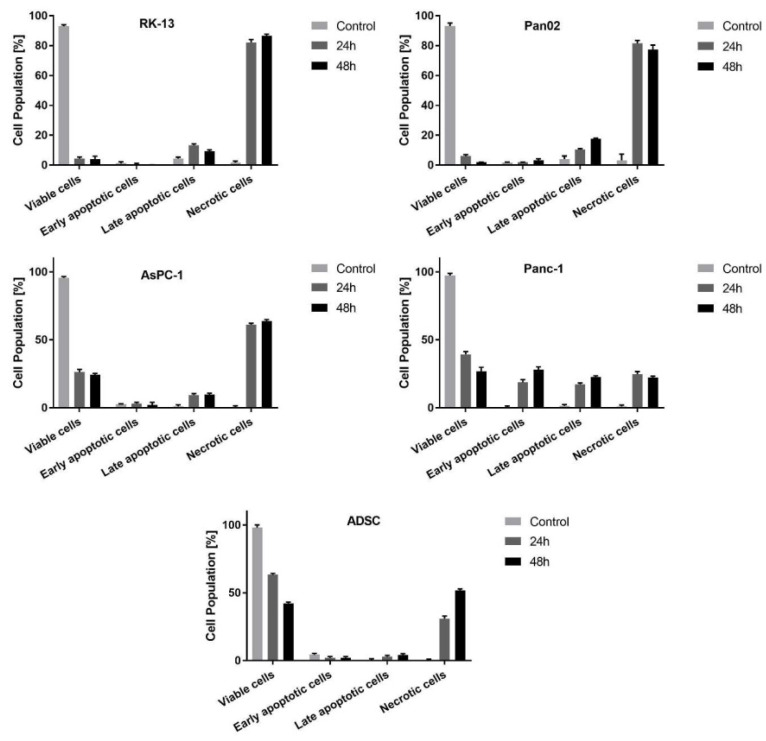
Examination of apoptosis and necrosis in cell lines: RK-13, Pan02, AsPC-1, Panc-1 and ADSCs infected with vMyx-LIGHT (MOI = 5) at 24 and 48 h after infection; flow cytometry, Annexin V (FITC channel) and 7-AAD (PerCP-Cy5.5 channel). The data show mean ± SD of two independent experiments.

Annexin-V and 7-AAD staining of cell cultures was performed to quantify early and late apoptosis and necrosis. Phosphatidylserine is expressed by early apoptotic cells, while the DNA of late apoptotic and necrotic cells can be stained with 7-AAD. After 24 and 48 h postinfection, RK13, Pan02 and AsPC-1 cell cultures showed significantly elevated numbers of necrotic cells (Figure 2 and Figure S2). Only Panc-1 cell cultures contained ca. 25% early and late apoptotic cells at those two time points p.i. Infected ADSCs retained significant viability at 24 and 48 h postinfection (63% and 42%, respectively). This result seems to corroborate the ability of ADSCs to function as an effective viral cargo carrier that is also capable of supporting viral replication and potentially delivering either parental or progeny viruses into cancer sites. 

### 3.3. In Vitro Determination of Optimized Gemcitabine Dose and Synergistic Effect in Combination with vMyx-LIGHT

**Figure 3:**
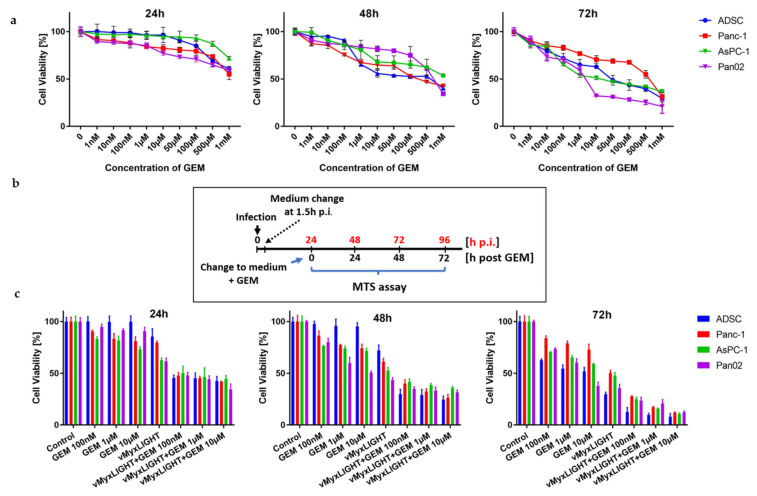
Viability of ADSC and pancreatic cancer cells after GEM treatment. (**a**) Determination of optimized GEM doses. Cell lines: ADSCs, Pan02, Panc-1, AsPC-1 treated with various GEM concentrations for 24, 48 and 72 h and analyzed for cell viability using MTS. (**b**) Timeline of MYXV and GEM experimental combination. (**c**) Combination of MYXV infection and GEM. Pancreatic cancer cells (Panc-1, AsPC-1 and Pan02) and ADSC (1 × 10^4^ cells/well) were infected with vMyx-LIGHT at MOI = 5 and/or treated with GEM and then analyzed for cell viability using MTS assay at 24, 48 and 72 h post-GEM treatment. The assays were performed in triplicate; error bars shown are mean ± SD.

We first optimized the smallest effective dose of commercial gemcitabine and assessed the viability of the three studied pancreatic cancer cell lines using three drug exposure times and concentrations ranging from 1 nM to 1 mM. All four cell lines were confirmed to be sensitive to gemcitabine (Figure 3a). At 72 h post-GEM addition, at 10 µM concentrations and higher, we found Pan02 cells to be ca. 40% viable, whereas the two human lines AsPC-1 and Panc-1 were ca. 50% and 80% viable, respectively. Of note, under these conditions, ADSC carrier cells retained approx. 63% viability. Based on these results, we selected three concentrations of GEM (10 µM, 1 µM and 100 nM) for further experiments. 

Next, we studied the possible synergy of cytotoxic effects using the cell cultures of three pancreatic cancer cell lines and ADSCs, exposed to LIGHT-armed MYXV construct (MOI = 5) followed by the addition of GEM at the three previously determined drug concentrations. GEM was added 24 h postinfection of cell cultures with the LIGHT construct (Figure 3b). 

The combined treatment (Figure 3c) resulted in the strengthening of the cytotoxic effect for all three GEM concentrations chosen. The viability of the tested cell lines, as measured by the MTS assay, was reduced by the 24 h time point to less than 50% and dropped down further by more than two-fold (to ca.15%) by 72 h.

### 3.4. Therapeutic Effect of ADSC-Shielded LIGHT-Armed MYXV Construct and Gemcitabine Combination on Induced Orthotopic PDAC Tumors In Vivo 

We previously found that implanting Pan02 cells (1 × 10^6^) in murine pancreata via abdominal surgery resulted in the establishment of sizeable orthotopic PDAC lesions within three weeks. All therapeutic manipulations were thus performed within this three-week period starting with oncolytic therapy on day 4 post engraftment of Pan02-luc cells (based on the level of bioluminescence, as measured by IVIS; see Figure S1). 

**Figure 4:**
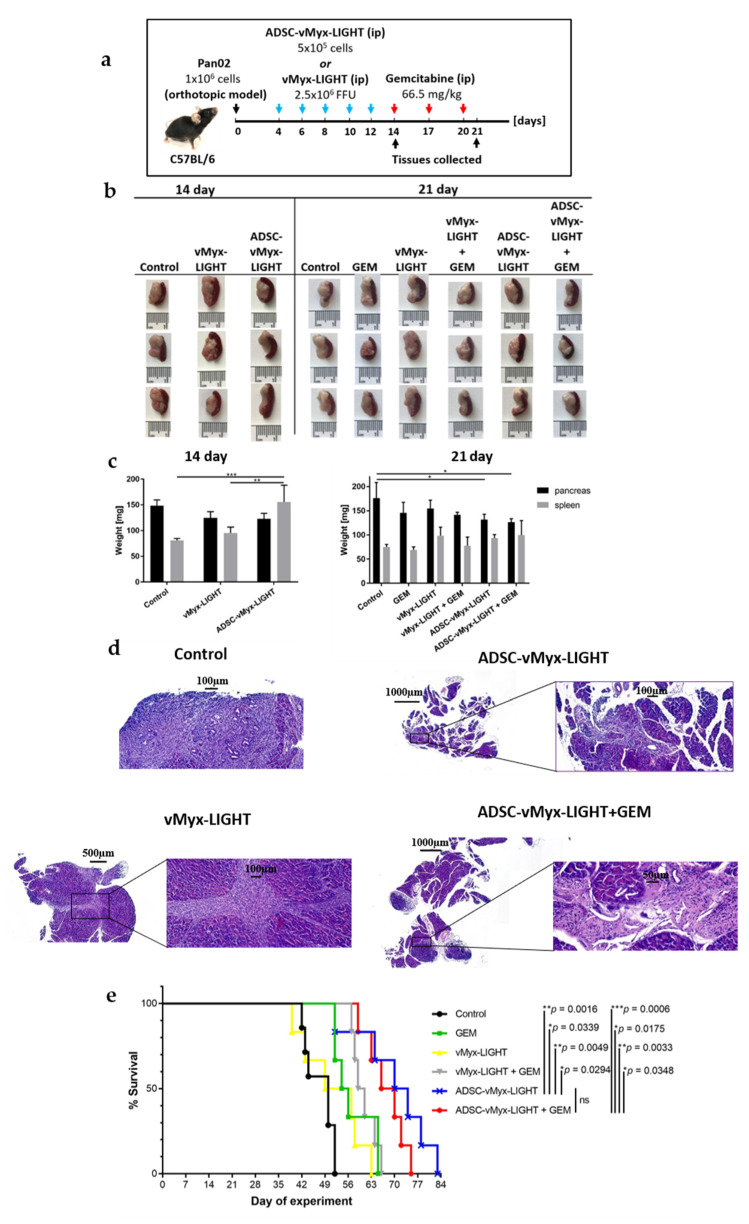
Combined therapy of experimental pancreatic adenocarcinoma using armed MYXV construct. C57Bl/6NCrl mice (*n* = 12) with induced orthotopic lesions were injected ip. (days 4, 6, 8, 10 and 12) with LIGHT-expressing MYXV—either ADSC-shielded (5 × 10^5^ cells/100 µL PBS¯) or unshielded (2.5 × 10^6^ FFU/100 µL PBS¯), and were then injected ip. (days 14, 17 and 20) with GEM (66.5 mg/kg in 100 µL PBS¯). (**a**) Timeline of experiment; (**b**) size and (**c**) weight of pancreata and spleens on days 14 and 21 (*n* = 3); (**d**) representative micrographs of H&E-stained sections derived from the indicated treatment groups (scale bars: 50–1000 µm); (**e**) mouse survival (*n* = 6): log rank test (Mantel–Cox). The data (mean ± SD) were analyzed with one-way ANOVA; statistically significant differences are indicated (* *p* ≤ 0.05; ** *p* ≤ 0.01; *** *p* ≤ 0.001).

Using this orthotopic PDAC model in immunocompetent mice, we examined the therapeutic outcome of treatment with the LIGHT-expressing oncolytic MYXV construct (vMyx-mLIGHT-Fluc/tdTr) alone or in combination with GEM (Figure 4a). For monotherapy, a total of five doses of unshielded (virus alone) or shielded (virus + ADSCs) vMyx-LIGHT was administered every 2 days (groups: vMyx-LIGHT and ADSC-vMyx-LIGHT). For combination therapy, three doses of GEM were injected ip. every 3 days following a 48 h break period after the conclusion of oncolytic virotherapy (groups: GEM, vMyx-LIGHT+GEM and ADSC-vMyx-LIGHT+GEM). Some of the treated mice were sacrificed in order to collect tissue material (pancreata, spleens and blood samples). The procedures were performed at two time points: after the conclusion of oncolytic therapy (day 14) and after the conclusion of gemcitabine administration (day 21). The excised pancreata and spleens were inspected for size and organ weight; part of the collected material (also including blood aliquots) was processed further for the histopathology assessment and verification of possible immune response triggered by therapy (see further). Compared to the control, no statistical difference was noted in the weight of pancreata following oncolytic therapy alone (day 14), either with the ADSC-shielded or with the unshielded myxoma construct (Figure 4b,c); spleens, to the contrary, were significantly heavier in the shielded groups, compared to both the control (*p* = 0.0003) and to the unshielded virus (*p* = 0.0018) groups. At the conclusion of therapy (day 21), slightly lighter pancreata were recorded for both mono- and combination therapy groups that received the ADSC-shielded virus, but only when compared to the control (*p* = 0.0482 and *p* = 0.0209, respectively). The weights of spleens at this time point were almost the same for all groups. Together, the pattern of size and weight changes seen in pancreata and spleens following the conclusion of therapy is suggestive of a positive response to both unshielded and ADSC-shielded forms of the oncolytic construct, with no clear difference between the two. 

H&E-stained pancreas tissue specimens from all treatment groups and controls were evaluated microscopically (Figure 4d) by an experienced histopathologist. Pancreatic specimens revealed strong fibrosis (untreated mice), while rich cell infiltration was present in specimens from the group treated with unshielded virus. In turn, pancreatic specimens from mice treated with the shielded virus, as well as specimens from mice treated with the shielded virus and gemcitabine showed even stronger lymphocytic infiltrates and also fibrosis. 

Survival extension was monitored for part of the experimental therapy cohort (Figure 4e). The differences in survival found between the control and both the shielded viral construct and shielded viral construct plus gemcitabine were significant (** *p* = 0.0016 and *** *p* = 0.0006, respectively), highlighting the benefits of using ADSCs for therapy. The survival extension of mice receiving unshielded virus, or gemcitabine only, or unshielded virus plus gemcitabine, was inferior compared to groups where the shielded construct was applied, suggestive of a quicker clearance of the oncolytic therapeutic. Strikingly, two of these groups where gemcitabine was used revealed a sharp drop in animal survival, suggesting an unexpected detrimental effect of gemcitabine. Additionally, an unexpected result was the slightly better survival of mice receiving ADSC-shielded virus but no gemcitabine as compared to mice that received ADSC-shielded virus plus the drug. This amplifies the previous suggestion concerning an unwelcome effect of gemcitabine in the experimental setting. Of note, on day 65 of therapy, when no more mice that received gemcitabine only were alive, the survival of mice which, prior to the chemotherapeutic, received ADSC-shielded virus was extended by 50%, as compared to 14% for mice that first received the unshielded virus. 

### 3.5. Assessment of Antitumor Response 

**Figure 5:**
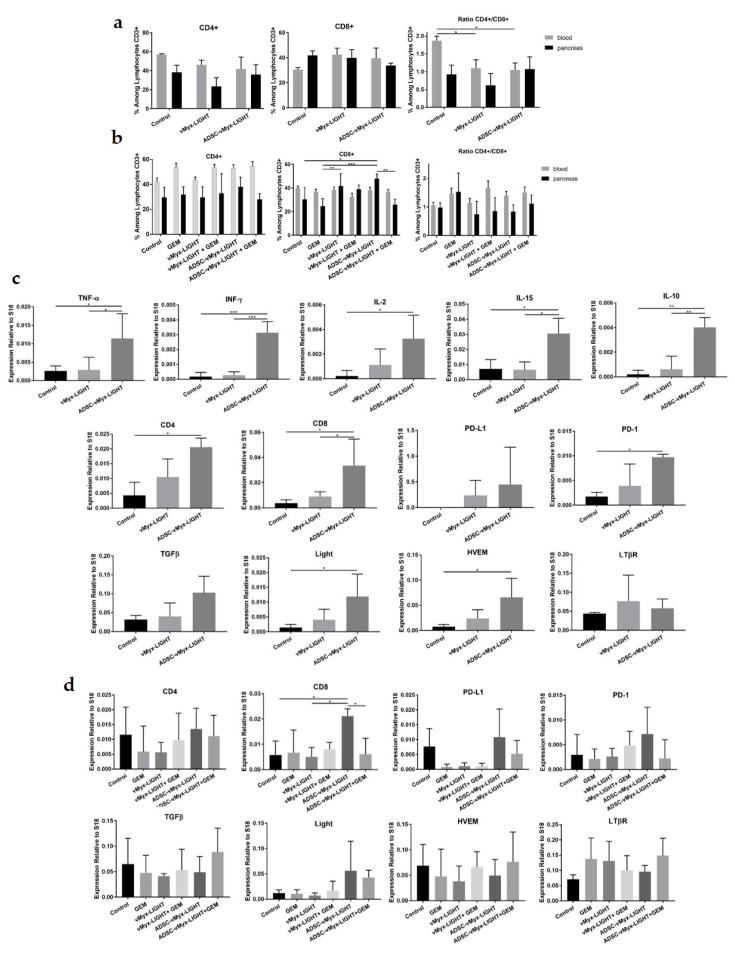
Antitumor immune response after combined therapy of experimental pancreatic adenocarcinoma using LIGHT-armed MYXV construct and GEM. C57Bl/ C57Bl/6NCrl mice (*n* = 6) with induced orthotopic lesions were injected ip. (days 4, 6, 8, 10 and 12) with LIGHT-expressing MYXV—either ADSC-shielded or unshielded; then, they were injected ip. (days 14, 17 and 20) with GEM. (**a**) Flow cytometry data (*n* = 3) showing CD4+ and CD8+ cell percentage among CD3+ lymphocytes in blood and pancreas on days 14 and (**b**) 21; (**c**) analysis of gene expression (RT-qPCR) in pancreata for: TNFα, INFγ, IL-2, IL-15, IL-10, TGF-β, CD4, CD8, PD-L1, PD-1, LIGHT, HVEM and LTβR (day 14) and (**d**) for: CD4, CD8, PD-L1, PD-1, TGF-β, LIGHT, HVEM and LTβR (day 21). Changes in the gene expression were rendered as a ratio of target gene vs. reference gene (S18) relative to expression in control samples. The data (mean ± SD) were analyzed with one-way ANOVA; statistically significant differences are indicated (* *p* ≤ 0.05; ** *p* ≤ 0.01; *** *p* ≤ 0.001).

As mentioned above, the antitumor immune response was assessed both after the conclusion of oncolytic therapy (day 14) and after combination therapy with gemcitabine (day 21). The material obtained from the collection of pancreata and blood samples was used for flow cytometry studies, and for RNA isolation and subsequent RT-qPCR. Samples of pancreatic tissue and blood aliquots examined by flow cytometry for signs of an adaptive anti-tumor immune response (Figure 5a,b) revealed the percentage of CD4+ and CD8+ among CD3+ lymphocytes to be unchanged on day 14, i.e., following the conclusion of oncolytic construct administration. At this time point, changes in the CD4+/CD8+ ratio were significant for both the shielded and unshielded virus groups, but only in the blood samples (Figure 5a). After the conclusion of combination therapy (day 21), an increase was noted in the CD8+ content of pancreatic samples derived from the shielded virus group, when compared to both the control and GEM groups (Figure 5b), as well as for unshielded virus but compared only to the GEM group. The CD8+ content was also increased in the shielded virus group in comparison to the shielded construct group that additionally received gemcitabine. No changes were found in the percentage of CD4+ helper cells nor in the CD4+/CD8+ ratio for any group.

Data for the gene expression of immune response-related cytokines in pancreatic specimens from mice that received 5-dose virotherapy revealed a clear difference between the groups treated with unshielded and shielded viral constructs. After the conclusion of oncolytic therapy (day 14), the shielded virus treatment group showed an increased expression of innate response-mediating pro-inflammatory cytokines, such as tumor necrosis factor alpha (TNF-α), interferon gamma (IFNγ), Interleukin-2 (IL-2) and Interleukin-15 (IL-15), as compared to unshielded MYXV, as well as the control groups (Figure 5c). The gene expression of IL-10, a cytokine with a direct effect on the maturation and differentiation of T cells, was upregulated in the group treated only with the shielded oncolytic construct (Figure 5c). The expression of TGF-β, another anti-inflammatory cytokine, was also found to be upregulated (Figure 5c), but this was not statistically significant. This cytokine can suppress or promote tumor growth, depending on the stage of tumor development, and can be produced by cancer cells, as well as by stromal cells. The TGF-β expression level appears to be of special importance in the context of oncolytic virotherapy, especially when its combination with gemcitabine is considered. 

The expression of genes that are markers for both CD4+ and CD8+ immune effector T cells was upregulated upon the conclusion of oncolytic therapy, on day 14 of the experiment (Figure 5c). On day 21, after the conclusion of combined therapy, the CD8+ marker was significantly upregulated for the shielded viral construct group, as compared to the group that received gemcitabine in addition to the shielded virus (Figure 5d). This finding underscores the activation of antitumor immune response in the wake of oncolytic therapy. The up-regulation of PD-1 and its ligand, PD-L1 (Figure 5c,d), was only significant on day 14 for PD-1, suggesting the possible susceptibility of PDAC to the use of checkpoint inhibitors (with the additional caveat of controlling the ablation of TGF-β). See also the Discussion and Supplementary Figure S3. 

Finally, the expression of genes encoding lymphotoxin-β receptor (LTβR) and herpes virus entry mediator (HVEM) was examined. LIGHT, the executive arm of the oncolytic construct used herein, can regulate the activity of T lymphocytes via binding to these receptors. We found the expression of the LIGHT gene and HVEM to be significantly upregulated upon the conclusion of virotherapy with the shielded virus, on day 14 of the experiment (Figure 5c). However, on day 21, more than a week after concluding the administration of the viral construct, the upregulation of the LIGHT gene in the shielded construct group was no longer statistically significant (Figure 5d).

## 4. Discussion

Patients who progress while receiving chemotherapy are a grim testimony of limited successes achieved by the current treatment of pancreatic cancer. However, combinations of emerging tools and traditional modalities do raise hope for a breakthrough. A case in point is oncolytic virotherapy in combination with chemotherapy. We previously reported the experimental treatment of murine PDAC using a second-generation genetically engineered oncolytic myxoma virus armed with *LIGHT* (*TNFSF14*) transgene. LIGHT is a protein playing a significant role in inducing the apoptosis of cancer cells and enhancing the immune modulation of the tumor microenvironment via the promotion of the tumor vessel normalization and generation of tertiary lymphoid structures [25]. The delivery, or induced presence, of LIGHT in the tumor microenvironment can create synergy with other agents that trigger and/or support immune reactions ultimately resulting in the enhanced recruitment of effector T lymphocytes, NK cells and reduced immunosuppression. However, systemic LIGHT overexpression can have possible autoimmune consequences [26,27,28]. Therefore, even though the potential of LIGHT as an immunotherapeutic anticancer agent has been explored for over a decade [29] in both primary and metastatic settings using various delivery systems, the specifics of pancreatic cancer would add additional difficulty to the targeting of the LIGHT-armed vector. Since mesenchymal stromal cells can migrate towards the inflammatory microenvironment and engraft into tumor stroma after systemic administration [30], we assumed that adipose tissue-derived mesenchymal stem cells (ADSCs) could both protect the viral cargo during transit to pancreatic sites and also enable passive targeting to essentially inflammatory intra- and peritumoral PDAC lesions [31]. We previously demonstrated that the shielding of this replication-competent LIGHT-armed oncolytic construct by ADSCs, achieved through ex vivo loading, can improve the outcome of PDAC treatment by reducing tumor burden. The effective transit, delivery and timely release of oncolytic viral particles in PDAC lesions require a therapeutically satisfactory time window, and we previously documented ADSCs to be sufficiently permissive to MYXV infection, yet robust enough to function as “viral factories”. The endurance of human ADSCs infected with therapeutic LIGHT-armed myxoma virus following ip. injection into immunocompetent mice was found to be sufficient to secure the survival of virus cargo during transit and its delivery to PDAC lesions before immune clearance [11].

Concerning chemotherapy, it has been shown that defects in apoptotic pathways and the deregulation of apoptotic proteins (such as PARP1 and AIF) are crucial for the development of PDAC, which is notoriously resistant to apoptosis [19]. To enhance the apoptosis of pancreatic cancer cells, oncolytic viruses (e.g., herpes simplex or adenovirus) armed with suicide or tumor suppressor genes have been used in conjunction with chemotherapy [32]. We here examined the capacity of the LIGHT-armed myxoma virus to yield the postulated synergistic effect when used in combination with gemcitabine, a gold standard drug used in PDAC chemotherapy. Known common side effects of this drug include bone marrow suppression (myelotoxicity), resulting in a decrease in the production of leukocytes. Gemcitabine can trigger apoptosis in pancreatic carcinoma cells by lowering the expression of Bcl-2 and activating caspases; it can also induce reactive oxygen species and block cell cycle in the S phase [33]. 

A decade ago, Wennier et al. [17] reported the use of MYXV in combination with gemcitabine for the treatment of experimental pancreatic cancer in mice employing a late-stage intraperitoneal-disseminated (IPD) tumor model and showed that the outcome critically depends on the order and timing strategy of administering both agents. The improved long-term survival of the treated immune competent mice was observed only when gemcitabine administration followed that of the oncolytic virus, suggesting intact immune response to be critical for survival extension. We wondered whether the curative outcome shown by Wennier et al. could be improved when using a more stringent orthotopic PDAC model based on surgically induced Pan02 lesions in the murine pancreas. Our intent was to analyze combination therapy in a model that better reproduces actual PDAC characteristics, particularly, its dense stroma composed of collagen-rich extracellular matrix, pancreatic stellate cells and inflammatory cells. The delivery of the oncolytic MYXV construct using a protective carrier seemed a rational choice to target the highly immunosuppressive desmoplastic tumor microenvironment of PDAC, as the antiviral response of the immune competent host can restrict the time window of active viral replication in tumors to the first week following virus injection [34,35]. 

First, we checked in vitro the ability of the LIGHT-armed MYXV construct to infect and replicate in the tested PDAC cell lines; then, we demonstrated the carrier ADSCs to effectively transfer the oncolytic cargo to cultured pancreatic cancer cells. FACS analysis revealed a necrotic-like type of cell death among murine Pan02 and human PDAC cell cultures exposed to LIGHT-armed MYXV. Pan02 cells were subsequently used to establish PDAC lesions in the pancreas of immunocompetent mice. MYXV-infected ADSCs retained significant viability even 24–48 h postinfection, confirming their status as an effective viral carrier. Although differences in the level of late viral protein (tdTr) expression among the infected cancer cell lines were suggestive of the variable permissiveness of these lines, the detected levels of early viral gene expression were deemed sufficient to warrant further in vivo studies. Gemcitabine cytotoxicity assessment in the examined pancreatic cancer cell cultures allowed us to demonstrate clear synergistic effects in vitro caused by treatment, first with the oncolytic myxoma construct and then (after 24 h) via exposure to gemcitabine. Using the combined treatment approach, we found the viability of tested cultured PDAC cells to decrease drastically (to ca. 15% at 72 h for Pan02 cells). Of note, at the concentration of GEM subsequently used for in vivo studies (10 µM), the viability of ADSCs carrier cells still remained at above 60%. 

For therapeutic experiments in vivo, we used immune competent mice with orthotopically induced Pan02 lesions; this particular cell line is highly sensitive to GEM [17] and permissive to MYXV infection. All therapeutic manipulations reported herein were performed within three weeks from the surgical inoculation of Pan02 cells. We thus examined the effect of oncolytic monotherapy using the LIGHT-expressing MYXV construct, either unshielded or shielded by ADSCs. For combined treatment, after concluding the oncolytic arm of the therapy, GEM was administered. The morphological aspects of the dissected pancreata and spleens suggested signs of early cellular immune response. 

The histopathology assessment of H&E-stained tissue specimens revealed cancer cell infiltrates particularly in the pancreata from the control group, whereas lymphocytic infiltrates were found in specimens from all groups, the strongest in those treated with the ADSC-shielded virus and weaker in specimens treated with unshielded virus. Taken together, the microscopic assessment suggests that the use of LIGHT-armed myxoma virus led to the modulation of the immune microenvironment in PDAC tissues. 

Concerning survival, the therapy of PDAC with unshielded LIGHT-armed myxoma did extend animal survival, similarly to monotherapy with GEM, as well as the combination of both agents. However, the rather abrupt demise of animals two months into therapy with GEM alone, as well as with the combination, suggests the adverse effect of GEM rather than that due to the oncolytic construct used. GEM, used at concentrations equal to half of the maximum tolerated dose and employing a standard bolus schedule, seemed to compromise the adaptive immune response boosted by the therapy with vMyx-LIGHT in the studied model, thereby not conferring a distinctive survival benefit over the use of ADSC-protected vMyx-LIGHT alone. On the other hand, our experimental therapy, making use of ADSCs to shield the LIGHT-armed virus, significantly extended survival in both the monotherapy and combined therapy groups, confirming the advantage of the “Trojan horse” strategy. Engraftment into the tumor stroma, targeting and diminished anti-viral response of the host [36] are likely to contribute to the edge offered by the stem cell-mediated delivery of the oncolytic construct tested. Additionally, exploiting ADSCs as a “Trojan horse” turned out to be beneficial in terms of the increased expression of some pro-inflammatory cytokines (TNF-α, IFNγ, IL-2, and IL-15) in pancreatic tissues following oncolytic treatment. These cytokines play an important role in mediating the innate immune response, and a clear contrast noted in favor of the shielded virus group early during therapy (day 14) suggests the effective penetration of PDAC stroma and a more robust innate response to the tumor. However, a somewhat striking survival advantage in favor of the shielded virus-only monotherapy over the combination of shielded virus and GEM probably parallels the disadvantageous GEM effect mentioned above. Nevertheless, reproducing the therapeutic result reported by Wennier et al., who used a disseminated PDAC model, turned out to be unfeasible in the stroma-rich orthotopic setting that likely more closely mimics PDAC in the clinical setting. 

A possible explanation for this is suggested by some clues in our flow cytometry (FC) and RT-qPCR data. The adaptive anti-tumor immune responses suggested by our FC data appeared towards the conclusion of combination therapy (day 21). Increased levels of CD8+ T cells were noted in the pancreas of mice treated with shielded or unshielded virus monotherapy. In contrast, the lowest CD8 value was recorded in the group of mice treated with GEM alone, as well as in the group of mice treated with ADSC-vMyx-LIGHT + GEM. The lack of changes in the percentage of CD4+ helper T cells, as well as only minor changes in the CD4+/CD8+ ratio and its tendency to decrease among groups involving GEM administration, suggests that the elicited immune response seen in the treated mice was triggered only by the oncolytic construct. This may be explained, especially in our model, by the myelosuppressive effect of GEM.

The balance between pro- and anti-inflammatory cytokines within the PDAC tumor microenvironment is dynamic and reflects the cross-talk between cancer cells and the inflammatory network cells. Among the vast bulk of the studied cytokines, the anti-inflammatory IL-10 and TGF-β play major roles in PDAC [37]. Using RT-qPCR, we noted in our study the upregulated expression level of IL-10 gene in the pancreata samples derived from mice treated with the shielded LIGHT-armed construct. As the immunostimulatory ability of IL-10 is known to allow the expansion of tumor-resident CD8+ cells, our results suggest that IL-10 is indeed implicated in the maturation and differentiation of T cells in tumors of mice exposed to the oncolytic viral agent. The signaling pathway of TGF-β, another cytokine examined, is one of the twelve core pathways implicated in PDAC, and disabling it might be among the critical events in halting PDAC progression. TGF-β is produced by cancer cells, as well as stromal cells within the tumor microenvironment, where it mediates the interaction between stellate cells and cancer cells, as well as impedes the functional activation of CTLs. Depending on the stage of tumor growth, TGF-β can exert opposite and context-dependent effects, e.g., suppress or promote tumor growth [1]. In our studies, the expression of TGF-β gene in the pancreata of mice treated with unshielded virus construct was not upregulated. Dai et al., using a different tumor model and LIGHT-armed adenovirus, reported a decreased level of TGF-β [38]. Our data show that the use of the ADSC-shielded myxoma construct led to a marked early increase in TGF-β expression, which later decreased. The administration of gemcitabine has been regarded as consistently increasing = TGF-β associated signals [39], and the results of administering GEM in our combination therapy group seemed to confirm that. Since TGF-β has been considered a prominent target in PDAC treatment, the level of this cytokine appears to be important. It is likely that immunotherapies combined with GEM would fail to alter the PDAC course unless they included also the ablation of TGF-β signaling. In the setting of TGF-β signaling deficiency, GEM and anti-PD-1 in the combined treatment led to a robust CD8+ response, decreased tumor burden and enhanced survival [39]. A positive trend seen in our TGF-β gene expression data could thus be further amplified by the genetic ablation or pharmacological inhibition of TGF-β signaling. Our RT-qPCR data for the expression of genes encoding markers of infiltrating immune effector T cells showed the activation of the antitumor immune response. Although this response appeared to be counteracted by the concomitant upregulation of PD-1 and its ligand PD-L1, the addition of checkpoint inhibitors to the proposed PDAC treatment could mitigate this effect. Finally, we checked the expression levels of lymphotoxin-β receptor (LTβR) and herpes virus entry mediator (HVEM) since the transgene (LIGHT) used to arm the myxoma construct can regulate the activity of T lymphocytes via binding to these two receptors. Not unexpectedly, we found that the expression of LIGHT gene and HVEM was generally upregulated. 

The exact reason why gemcitabine might inhibit T-cell infiltration is not known. Gemcitabine only weakly penetrates into the PDAC desmoplastic TME. In contrast, myxoma virus and ADSCs (also loaded with virus) can penetrate tumor lesions; thus, the combined treatment should result in an improved oncolytic effect and facilitate the penetration of gemcitabine. Additionally, by virtue of eliminating myeloid-derived suppressor cells (MDSCs), gemcitabine should enhance OV efficacy [18]. We could not confirm this enhancement in our model. Gemcitabine seems to suppress T-cell infiltration into the tumor tissues, and this has been reported for animal models and clinical PDAC samples. Perhaps suboptimal doses of gemcitabine were used in our experiments. 

In sum, the use of LIGHT-armed myxoma oncolytic therapy combined with gemcitabine in an orthotopic PDAC model has its drawbacks, but the proposed oncolytic therapy can be improved and warrants further consideration. The refinements could include, inter alia, exploring the delivery of shielded/targeted GEM (or GEM lipophilic prodrug derivatives) [19], the use of cisplatin combinations [40] with checkpoint inhibitors [41], the implementation of anti-stromal strategy [42] and the genetic or pharmacological inhibition of TGF-β or its receptor signaling [43,44]. All of these might enhance the vironcolytic approach and lead to the functional activation of tumor-infiltrating lymphocytes, which perhaps could offer hope to progressing PDAC patients. 

## 5. Conclusions

Adjunct co-therapy in a stroma-rich model of orthotopic experimental murine PDAC based on an ADSC-shielded LIGHT-armed myxoma construct with the additional sequential administration of gemcitabine did not improve the results of oncolytic monotherapy. The level of CD8+ in pancreatic tissues confirmed both the antitumor immune response triggered by the oncolytic construct and showed the benefit from using ADSCs as a carrier for the viral construct. The lack of clear-cut therapeutic benefit in vivo from adding gemcitabine as a co-treatment is at odds with the synergy demonstrated against PDAC cells cultured in vitro. This could be due to the effect of desmoplasia presence, or GEM-induced myelotoxicity adversely affecting the immune arm of the oncolytic therapy, or both. Gemcitabine can significantly inhibit CD8+ T-cell infiltration, which was observed both in mouse tumor allografts and in human pancreatic cancer tissues [40]. The possible refinement of the therapy is laid out in the Discussion. 

## Data Availability

Data are maintained within this article and are not publicly available due to privacy.

## Supplementary Materials

The following supporting information can be downloaded at: https://www.mdpi.com/article/10.3390/cancers14082022/s1, Figure S1: Establishment of orthotopic pancreatic tumor, Figure S2: Quantitative Analysis of Apoptosis by Flow Cytometry, Figure S3: Antitumor immune response after monotherapy of experimental pancreatic adenocarcinoma using LIGHT-armed MYXV construct; Table S1: Primers.
