# Supplementary material for: Combination of LIGHT (TNFSF14)-Armed Myxoma Virus Pre-Loaded into ADSCs and Gemcitabine in the Treatment of Experimental Orthotopic Murine Pancreatic Adenocarcinoma
Source: Cancers (Basel). 2022 Apr 16;14(8):2022. doi: 10.3390/cancers14082022 (PMC9027757; doi:10.3390/cancers14082022)
Supplement: Supplementary file 1 [file cancers-14-02022-s001.zip › cancers-1647752-supplementary.pdf]

cancers MDPI

# Supplementary Materials: Combination of LIGHT(TNFSF14)-Armed Myxoma Virus Pre-loaded into ADSCs and Gemcitabine in the Treatment of Experimental Orthotopic Murine Pancreatic Adenocarcinoma

**Joanna Jazowiecka-Rakus, Aleksander Sochanik, Agata Hadryś, Wojciech Fidyk, Ewa Chmielik, Masmudur M. Rahman, Grant McFadden**

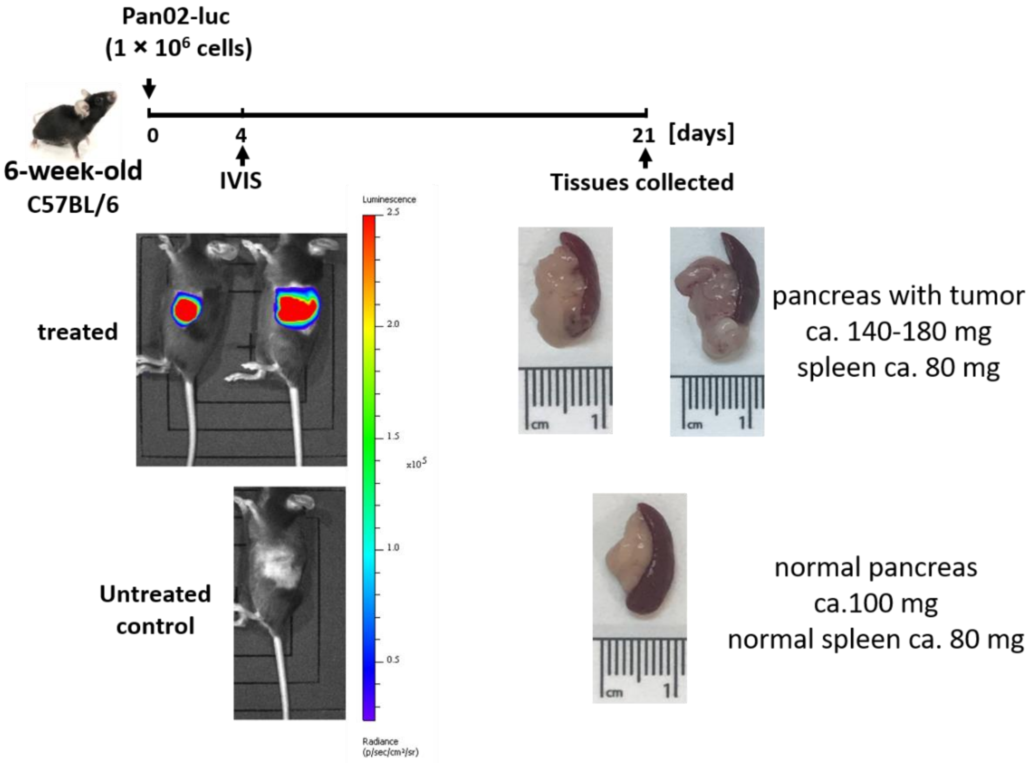


**Figure S1.** Establishment of orthotopic pancreatic tumors. C57BL/6 mice were orthotopically injected (day 0) with 1 × $10^6$ Pan02-luc cells suspended in 30 µL PBS$^-$. Four days after implantation, mice were anesthetized with 2.5% isoflurane and intraperitoneally injected with 1.5 mg D-luciferin (Promega) and bioluminescence images were acquired. BLI expressed as radiance (photons/sec/$cm^2$/sr). After 21 days the dissected organs (pancreata and spleens) were macroscopically examined for size as well as organ weight.

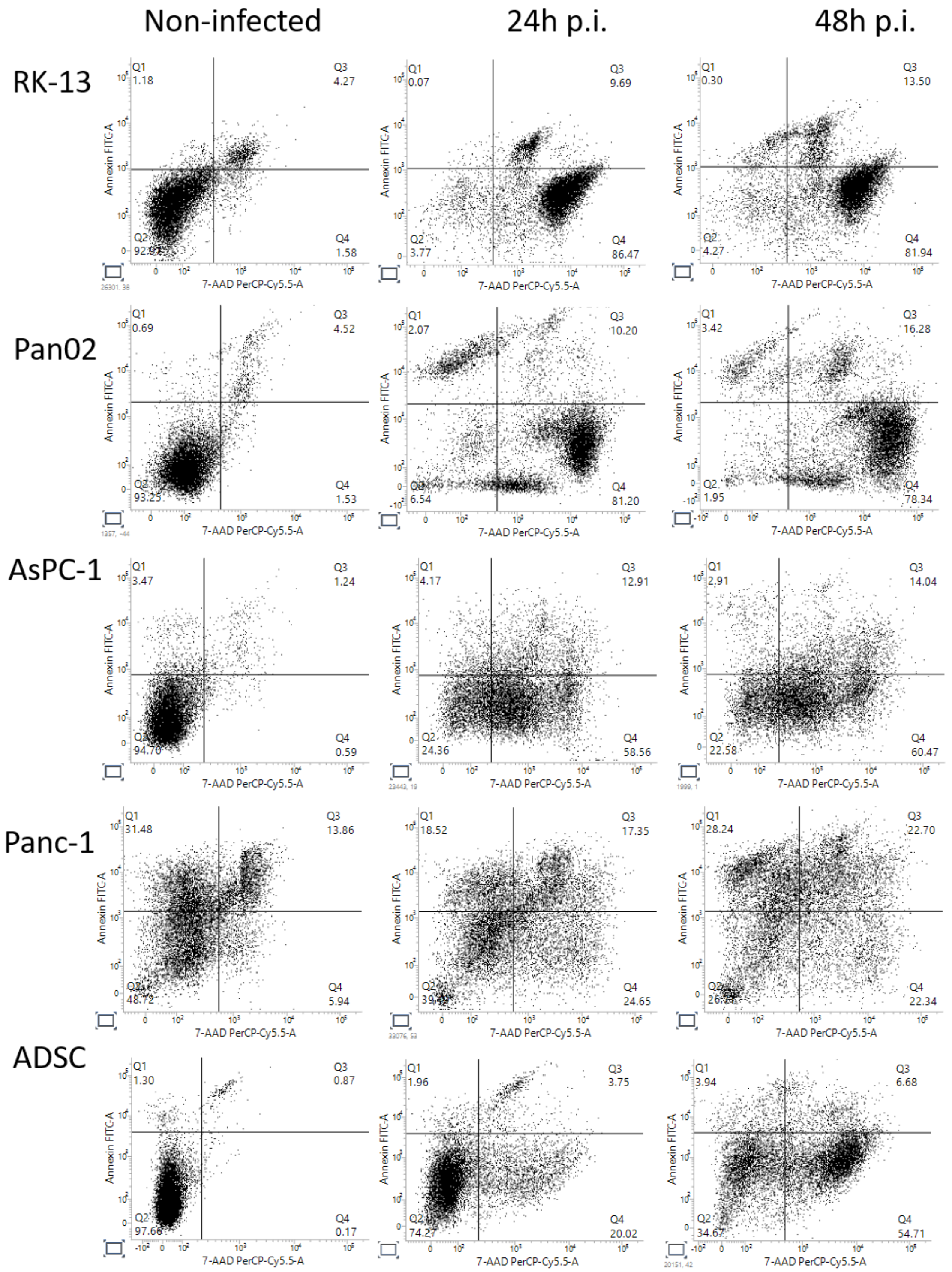


**Figure S2.** Quantitative Analysis of Apoptosis by Flow Cytometry. Cells were seeded at a density of 1 × $10^5$ cells/well using 6-well plates and vMyx-mLIGHT-FLuc/tdTr (MOI = 5) was added to the cultured cells. After 24 h and 48 h the cells were collected and washed twice with $PBS^-$ and staining buffer. Then the cells were stained with anti-Annexin V antibody and 7-aminoactinomycin D (BioLegend 640922) and analyzed for apoptosis/necrosis using flow cytometry (BD FACS Canto II). Annexin V was detected using FITC channel and 7-AAD using PerCP-Cy5.5 channel and a region for live cells was defined. Non-infected cells were used as a control. Flow cytometry plots for non-infected and infected with vMyx-LIGHT cell lines: RK-13, Pan02, AsPC-1, Panc-1 and ADSCs. Q1 - early apoptotic cells; Q2 - viable cells; Q3 - late apoptotic cells and Q4 - necrotic cells.

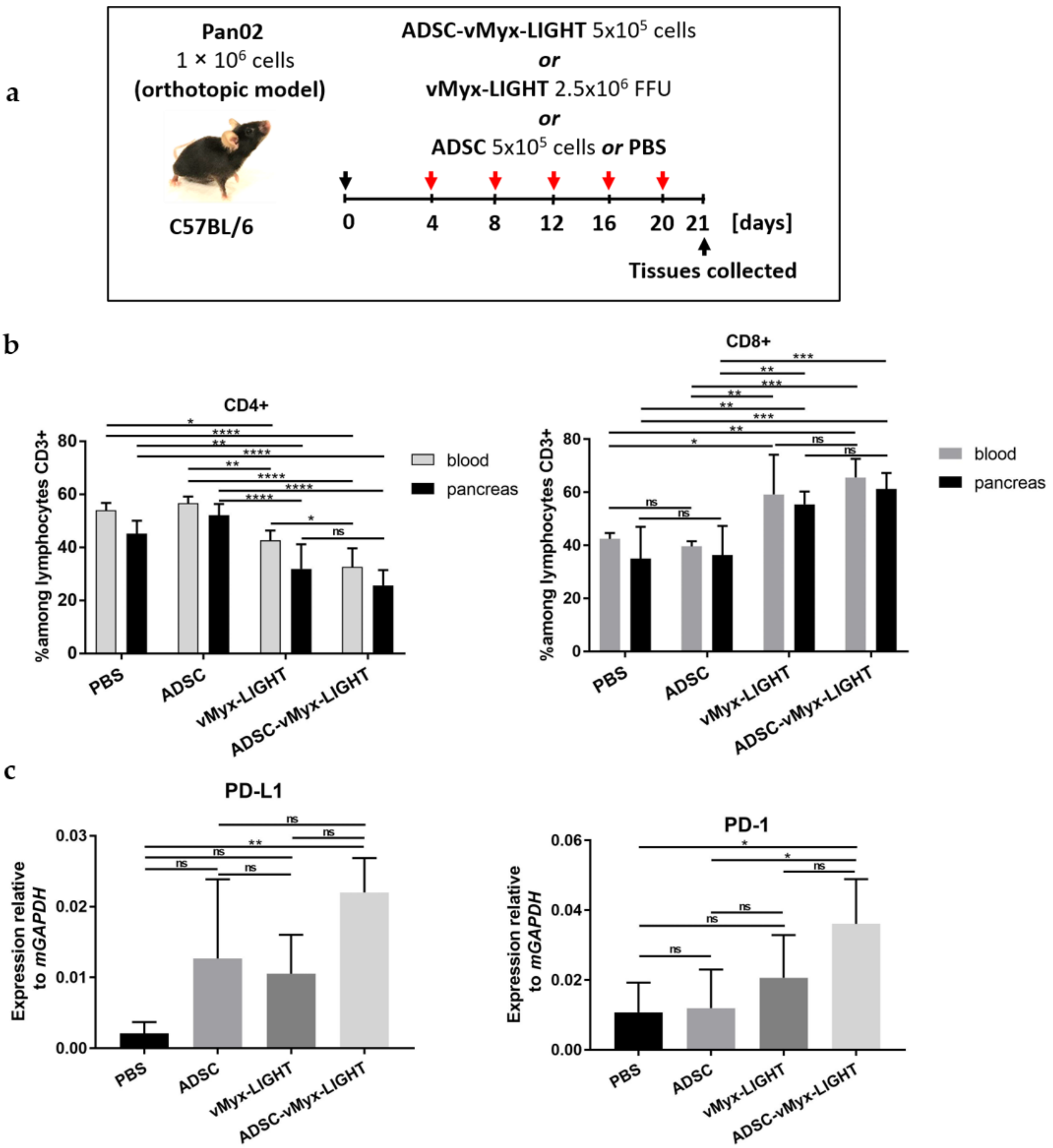


**Figure S3.** Antitumor immune response after monotherapy of experimental pancreatic adenocarcinoma using LIGHT-armed MYXV construct. Pancreatic tumors were established in recipient C57Bl/6NCrl mice ($n$ = 5/group) (day 0) by orthotopic implantation of 1 × $10^6$ Pan02 cells/30 µL $PBS^-$. For five-dose treatment regimens (days 4, 8, 12, 16 and 20), mice were injected IP with ADSCs (5 × $10^5$ cells/100 µL $PBS^-$) or ADSCs infected (MOI = 5) for 24 h with vMyx-LIGHT (5 × $10^5$ cells/100 µL $PBS^-$) or with unshielded vMyx-LIGHT (2.5 × $10^6$ FFU/100 µL $PBS^-$), or 100 µL $PBS^-$ (control). After 21 days, mice were sacrificed and the pancreata were excised. (**a**) timeline of experiment; (**b**) flow cytometry data showing CD4+ and CD8+ cell percentage among CD3+ lymphocytes in blood and pancreata (21st day); (c) Analysis of PD-L1 and PD-1 gene expression (RT-qPCR) in pancreata. Changes in the gene expression were rendered as a ratio of target gene vs. reference gene (*mGAPDH*) relative to expression in control samples. The data (mean ± SD) were analyzed with one-way ANOVA; statistically significant differences are indicated (*$p \leq 0.05$; ** $p \leq 0.01$; ***$p \leq 0.001$; ****$p \leq 0.0001$; ns: non-significant differences).

**Table S1.** Primers.

| Gene name | Forward primer (5'-3') | Reverse primer (5'–3') |
|---|---|---|
| *mTNF-α* | GGTGCCTATGTCTCAGCCTCTT | GCCATAGAACTGATGAGAGGGAG |
| *mINFγ* | CAGCAACAGCAAGGCGAAAAAGG | TTTCCGCTTCCTGAGGCTGGAT |
| *mIL2* | GCGGCATGTTCTGGATTTGACTC | CCACCACAGTTGCTGACTCATC |
| *mIL-15* | GTAGGTCTCCCTAAAACAGAGGC | TCCAGGAGAAAGCAGTTCATTGC |
| *mIL10* | CGGGAAGACAATAACTGCACCC | CGGTTAGCAGTATGTTGTCCAGC |
| *mTGFβ* | TGATACGCCTGAGTGGCTGTCT | CACAAGAGCAGTGAGCGCTGAA |
| *mCD8a* | ACTACCAAGCCAGTGCTGCGAA | ATCACAGGCGAAGTCCAATCCG |
| *mCD4* | GTTCAGGACAGCGACTTCTGGA | GAAGGAGAACTCCGCTGACTCT |
| *mPD-L1* | TGCGGACTACAAGCGAATCACG | CTCAGCTTCTGGATAACCCTCG |
| *mPD-1* | CGGTTTCAAGGCATGGTCATTGG | TCAGAGTGTCGTCCTTGCTTCC |
| *mLight* | GGAGACATAGTAGCTCATCTGCC | CCACCAATACCTATCAAGCTGGC |
| *mHVEM* | CCAGGCTACTTCTGTGAGAACC | CAGTCAGCACATACAGTGTCCTG |
| *mLTβR* | TCCTTGGAGGAAGTGGTGCTAC | CGGTCACATGAATGCCATTCGC |
| *S18* | CGGAAAATAGCCTTCGCCATCAC | ATCACTCGCTCCACCTCATCCT |
| *mGAPDH* | CATCACTGCCACCCAGAAGACTG | ATGCCAGTGAGCTTCCCGTTCAG |
| *hGAPDH* | GTCTCCTCTGACTTCAACAGCG | ACCACCCTGTTGCTGTAGCCAA |
